# Intraoperative Ultrasound Applications in Intracranial Surgery

**DOI:** 10.3389/fvets.2021.725867

**Published:** 2021-11-16

**Authors:** Andy Shores, Alison M. Lee, S. T. Kornberg, Chris Tollefson, Marc A. Seitz, R. W. Wills, Michaela J. Beasley

**Affiliations:** Department of Clinical Sciences, Mississippi State University College of Veterinary Medicine, Mississippi State, MS, United States

**Keywords:** intraoperative ultrasound, craniectomy, craniotomy, real-time imaging, veterinary

## Abstract

The methods and use of intraoperative ultrasound in 33 canine and five feline patients and its ability to localize and identify anatomical structures and pathological lesions in canines and felines undergoing intracranial surgery are described from a case series. All were client-owned referral patients admitted for neurologic evaluation, with an advanced imaging diagnosis of an intracranial lesion, and underwent surgical biopsy or surgical removal of the lesion. Medical records, retrieval and review of imaging reports, and characterization of findings for all canine and feline patients show that intraoperative ultrasound guidance was used in intracranial procedures during the period of 2012 and 2019. Twenty-nine of the canine patients had intracranial tumors. The remainder had various other conditions requiring intracranial intervention. Three of the feline patients had meningiomas, one had a depressed skull fracture, and one had an epidural hematoma. The tumors appeared hyperechoic on intraoperative ultrasound with the exception of cystic portions of the masses and correlated with the size and location seen on advanced imaging. Statistical comparison of the size of images seen on ultrasound and on MRI for 20 of the canine tumors revealed no statistical differences. Neuroanatomical structures, including vascular components, were easily identified, and tumor images correlated well with preoperative advanced imaging. The authors conclude that intraoperative ultrasound is a valuable asset in intracranial mass removals and can augment surgical guidance in a variety of intracranial disorders that require surgery. This is the first known publication in veterinary surgery of using intraoperative ultrasound as a tool in the operating theater to identify, localize, and monitor the removal/biopsy of intracranial lesions in small animals undergoing craniotomy/craniectomy.

## Introduction

The use of intraoperative ultrasound guidance for removal of intracranial masses in humans was first described by Rubin and Dohrman (1). Intraoperative ultrasound (IOUS) has since become an integral part of human intracranial surgery (2). Even in the face of advanced imaging and stereotactic techniques, the use of IOUS has flourished with the cited need of real-time imaging to identify the effects of intraoperative brain shift associated with pressure changes and changes occurring as the mass is removed. Recently, fusion of the ultrasound images with preoperative MRI has further advanced this technology, providing the neurosurgeon with a more complete view of the mass (2–4). In veterinary neurosurgery, Shores et al. presented the use of IOUS in 25 patients (5). Since that report, the use of IOUS has been continued by the authors and has become an essential component of intracranial surgery, especially in the removal of intracranial tumors.

## Materials and Equipment

Medical records database was searched for all patients that had intracranial IOUS, performed from 2011 to 2019. Thirty-eight patients were identified that had been admitted to the MSSTATE-CVM Animal Health Center's Neurosurgery/Neurology Service to evaluate a neurologic condition (Table 1) and subsequently underwent intracranial surgery and had IOUS guidance. Some patients had pre-existing advanced imaging and were admitted as tertiary referrals. Other patients were imaged after admission and a presumptive diagnosis made after magnetic resonance imaging (MRI)^1^ or computed tomography (CT)^2^. Images were reviewed by the radiologists at MSSTATE-CVM. Surgeries were performed using either a transfrontal, rostrotentorial, or suboccipital approach. All surgeries were performed by faculty in the MSSTATE-CVM Neurosurgery/Neurology Section. Anesthesia in all patients was performed by the anesthesia service at the MSSTATE-CVM and consisted of an intravenous protocol with ventilatory oxygen support. In general, majority of the procedures were performed with propofol induction followed by a remifentanil CRI. At the start of surgery, patients are paralyzed with cisatracurium and re-dosed as needed based on train-of-four testing at the stifle.

**Table 1:** Summary of patients undergoing craniotomy/craniectomy and intraoperative ultrasound (2012–2019).

**Case No**.	**Age (months)**	**Breed**	**Localization**	**Imaging**	**Approach**	**Diagnosis**
1	159	Chihuahua	C	MRI	Rostro	M
2	138	Yorkshire Terrier	C	MRI	Rostro	G-L
3	98	Pekingese-Poodle	C	MRI	Rostro	Other^a^
4	99	Collie	VCr	MRI	Rostro	Other^b^
5	126	Golden Retriever	PM	MRI	SO	M
6	60	Akita	C	MRI	TF	Other^c^
7	108	English Bulldog	C	MRI	TF	M
8	127	Labrador Retriever	CB	MRI	SO	M
9	156	Weimaraner	CB	MRI	SO	M
10	108	Labrador Retriever	C	MRI	Rostro	CPP
11	130	Labrador Retriever	C	MRI	Rostro	M
12	36	German Shorthair Pointer	C	MRI	Rostro	Other^d^
13	125	Boxer	C	MRI	Rostro	M
14	72	Labrador Retriever	C	MRI	TF	G-L
15	105	German Shepherd	C	MRI	Rostro	M
16	150	Terrier Mix	C	MRI	TF	M
17	96	Carin Terrier	C	MRI	Rostro	M
18	48	Cocker Spaniel	CB	MRI	SO	Other^e^
19	74	German Shepherd	C	MRI	Rostro	M
20	64	Boxer	CB	MRI	SO	G-H
21	15	Australian Cattle Dog	CB	MRI	SO	Other^f^
22	70	French Bulldog	C	MRI	Rostro	G-H
23	120	Golden Retriever	C	MRI	TF	M
24	125	Boxer	C	MRI	Rostro	G-H
25	144	Terrier Mix	C	MRI	Rostro	Other^g^
26	156	West Highland Terrier	C	MRI	TF	M
27	121	Chihuahua	C	MRI	TF	M
28	50	Carin Terrier	C	MRI	TF	M
29	121	Schnauzer	C	MRI	TF	M
30	127	American Bulldog	C	MRI	TF	M
31	70	English Bulldog	C	MRI	Rostro	Other^a^
32	83	King Shepherd	C	MRI	Rostro	G-H
33	132	Boston Terrier	C	MRI	Rostro	G-L
34	72	Domestic Short HairFeline	C	MRI	Rostro	M
35	84	Domestic Short HairFeline	C	CT	Rostro	M
36	8	Domestic Short HairFeline	C	MRI	Rostro	Other^h^
37	168	Domestic Short HairFeline	C	MRI	Rostro	M
38	60	Domestic Short HairFeline	C	MRI	Rostro	Other^i^

*Localization—C, Cerebral Cortex; CB, Cerebellar; VCr, Ventral Cranium; PM, Pontomedullary*.

*Approach—Rostro, Rostrotentorial; TF, Transfrontal; SO, Suboccipital*.

*Diagnosis—G-H, Glioma High-Grade; G-L, Glioma Low-Grade; M, Meningioma; CPP, Choroid Plexus Papilloma; ^a^Intraaxial Hematoma; ^b^Rathke's Cleft Cyst; ^c^Isolated Angiitis of the CNS; ^d^Vascular Anomaly; ^e^Epidermoid Cyst; ^f^Quadrigeminal Diverticulum; ^g^Granular Cell Tumor and Meningioma; ^h^Fracture/Porencephaly; ^i^Epidural Hematoma*.

The ultrasound units and the probes used to perform the intraoperative imaging are listed in the footnotes below^3^^,^^4^.

In this case series, all patients were referred for evaluation of clinical signs suggesting intracranial disease. Some of the patients were tertiary referrals and already with prior imaging that indicated the presence of intracranial disease. All patients had complete physical and neurologic examinations, three-view thoracic radiographs, and routine blood work (CBC, serum chemistries). Mild (grade 2/6) heart murmurs were noted in two patients but did not preclude anesthesia and surgery. Neurologic exam findings were variable and were either normal in patients presented only for intermittent seizure episodes or showed signs of typical of a left or right cerebral syndrome (circling ipsilateral to lesion, contralateral menace deficits, and contralateral limb postural deficits), cerebellar syndrome (three patients), and pontomedullary signs in one patient.

## Methods

**Figure 1:**
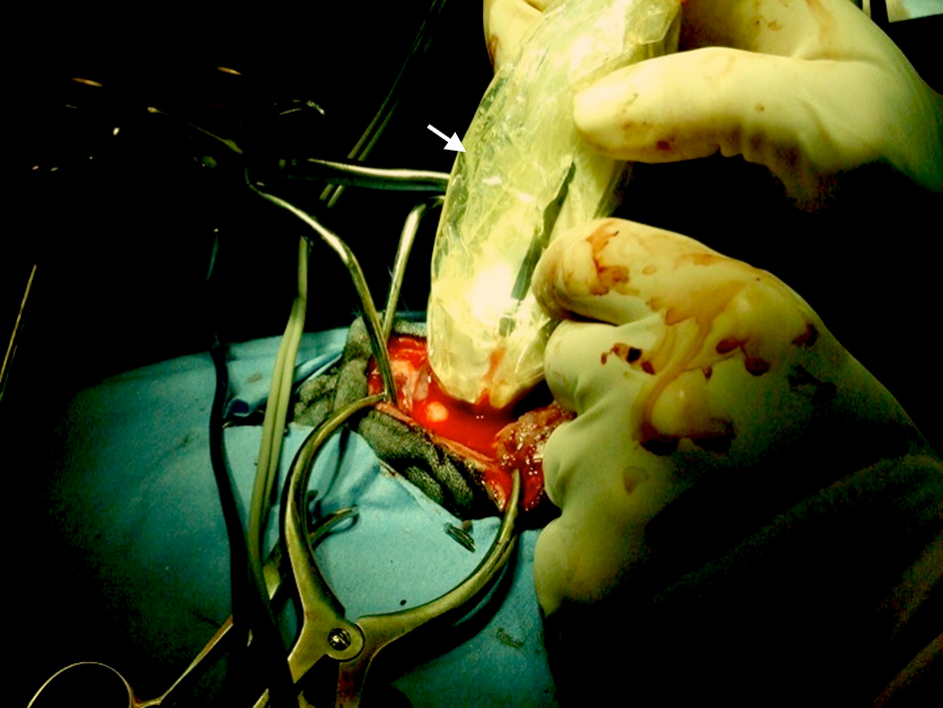
Intraoperative photo showing ultrasound transducer covered with a sterile sleeve (white arrow) and applied directly to the cerebral cortex during a rostrotentorial craniectomy.

After performing a transfrontal craniotomy or a rostrotentorial or suboccipital craniectomy and incising the dura with a #12 scalpel blade, we used intraoperative ultrasound to identify the mass or lesion^3^^,^^4^. The ultrasound probe (transducer) was covered in a commercially available sterile sleeve containing sterile ultrasound gel^5^. The covered transducer was applied directly to the cortex after filling the craniotomy/craniectomy defect with warmed sterile saline to provide improved acoustic coupling (Figure 1). A 5- to 8-MHz transducer was used and the IOUS images and the MRI or CT scans were available for the attending radiologist and neurosurgeons in the operating room at the time of all surgeries. Real time M-mode IOUS and Doppler IOUS imaging guided surgical resection using a Cavitron ultrasonic surgical aspirator (CUSA)^6^. Reports on IOUS imaging were completed by the radiology service to include descriptions of the size and imaging characteristics of the lesions.

Data were obtained from 20 of the canine tumors (Table 2) to evaluate height and width of the masses in a transverse plane for both MRI and IOUS. The height, width, and cross-sectional area (product of the height and width) were used to compare MRI and IOUS imaging of the masses. Statistical analysis (6) of each was compared using paired *t*-tests with PROC TTEST^7^.

**Table 2:** Measurement comparison of MRI and IOUS images for 20 canine patients.

**Case no**.	**U/S measurement (cm)**	**MRI measurement (cm)**	**U/S product**	**MRI product**
1	1.93 × 1.63	1.96 × 1.65	3.15	3.23
9	1.08 × 1.25	1.07 × 1.20	1.35	1.28
10	1.14 × 1.72	1.14 × 1.80	1.96	2.05
11	0.73 × 1.10	0.74 × 1.00	0.8	0.74
12	0.82 × 1.17	0.81 × 0.91	0.96	0.74
13	1.01 × 1.45	0.93 × 1.54	1.46	1.43
14	2.12 × 1.48	2.13 × 1.41	3.14	3
15	1.40 × 1.20	1.37 × 1.20	1.68	1.64
16	1.77 × 1.34	1.74 × 1.4	2.37	2.43
17	1.94 × 1.43	1.94 × 1.45	2.77	2.81
19	1.04 × 1.06	1.08 × 1.08	1.1	1.17
21	1.47 × 1.49	1.42 × 1.53	2.19	2.19
22	1.13 × 0.89	1.03 × 0.88	1	0.91
23	2.33 × 1.95	2.13 × 1.95	4.54	4.15
24	1.75 × 1.83	1.65 × 1.80	3.2	2.97
25	2.50 × 1.73	2.46 × 1.70	4.32	4.18
26	1.51 × 1.21	1.58 × 1.25	1.83	1.98
27	1.7 × 1.64	1.70 × 1.62	2.79	2.75
28	1.45 × 1.01	1.43 × 0.98	1.46	1.4
29	1.49 × 0.85	1.46 × 0.81	1.27	1.18

## Results

**Figure 2:**
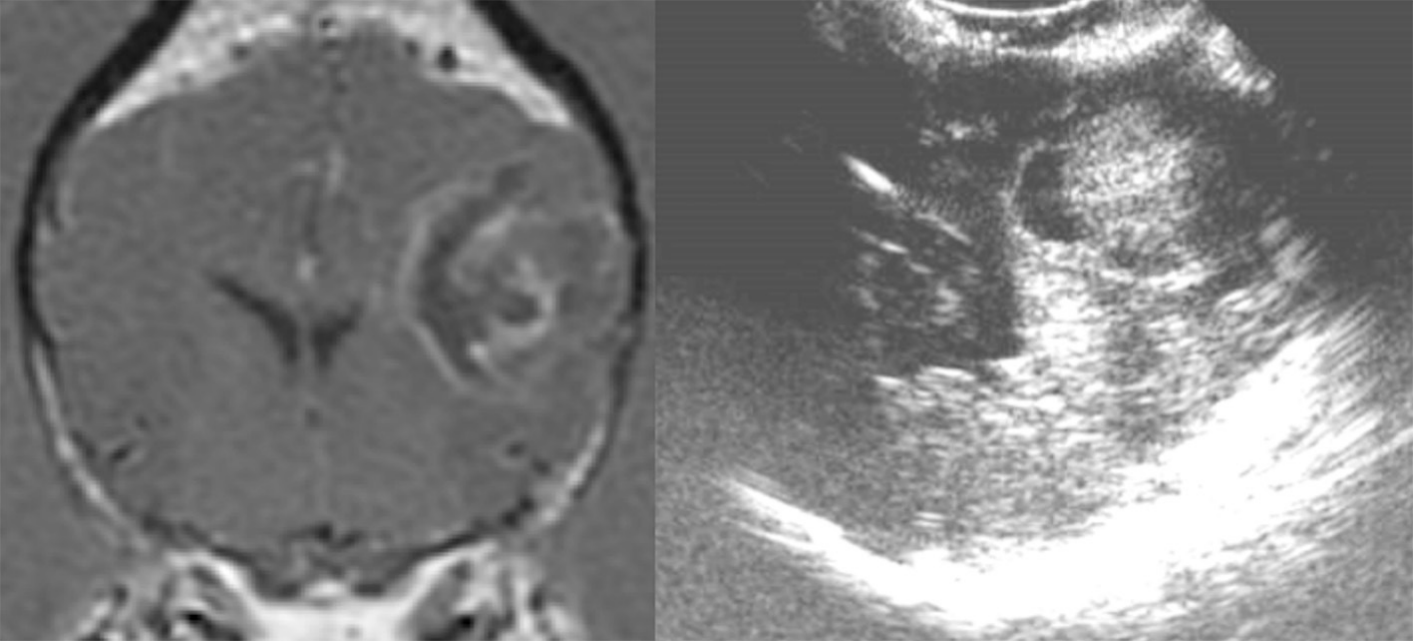
Case 24. There is an ovoid, well-circumscribed, 1.65 cm by 1.80 cm (MRI)/1.83 × 1.75 cm (US), heterogeneous, centrally hyperechoic and rim hypoechoic mass present in the right cerebrum, which results in medial displacement of the ultrasonographically normal brain parenchyma. There are a few pockets of anechoic fluid present medial and caudal this mass. This mass was a high-grade oligodendroglioma.

**Figure 3:**
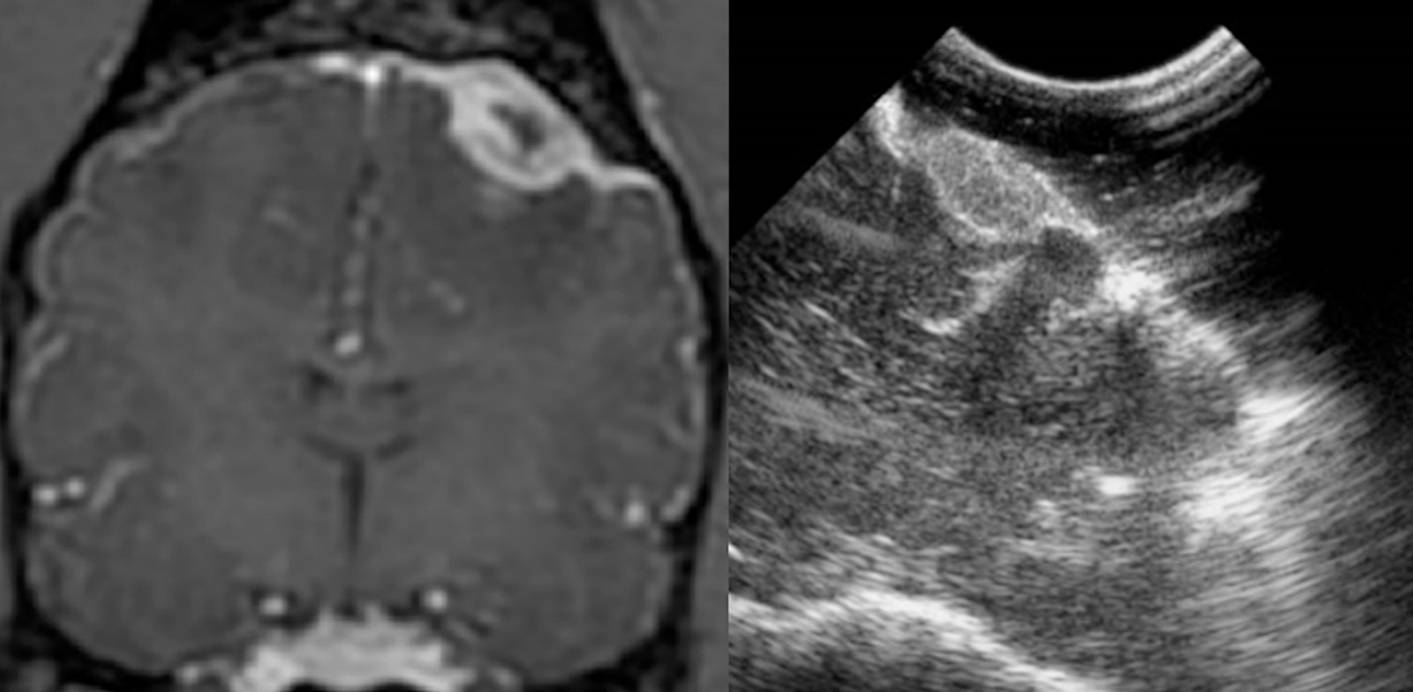
Case 13. There is a small, 0.74 × 1.0 cm (MRI)/0.73 × 1.1 cm (US), ovoid, well-circumscribed, heterogeneous mass present in the periphery of the parietal lobe, which is hyperechoic to the remaining brain parenchyma and displays a strongly hyperechoic rim. This mass was a meningioma.

**Figure 4:**
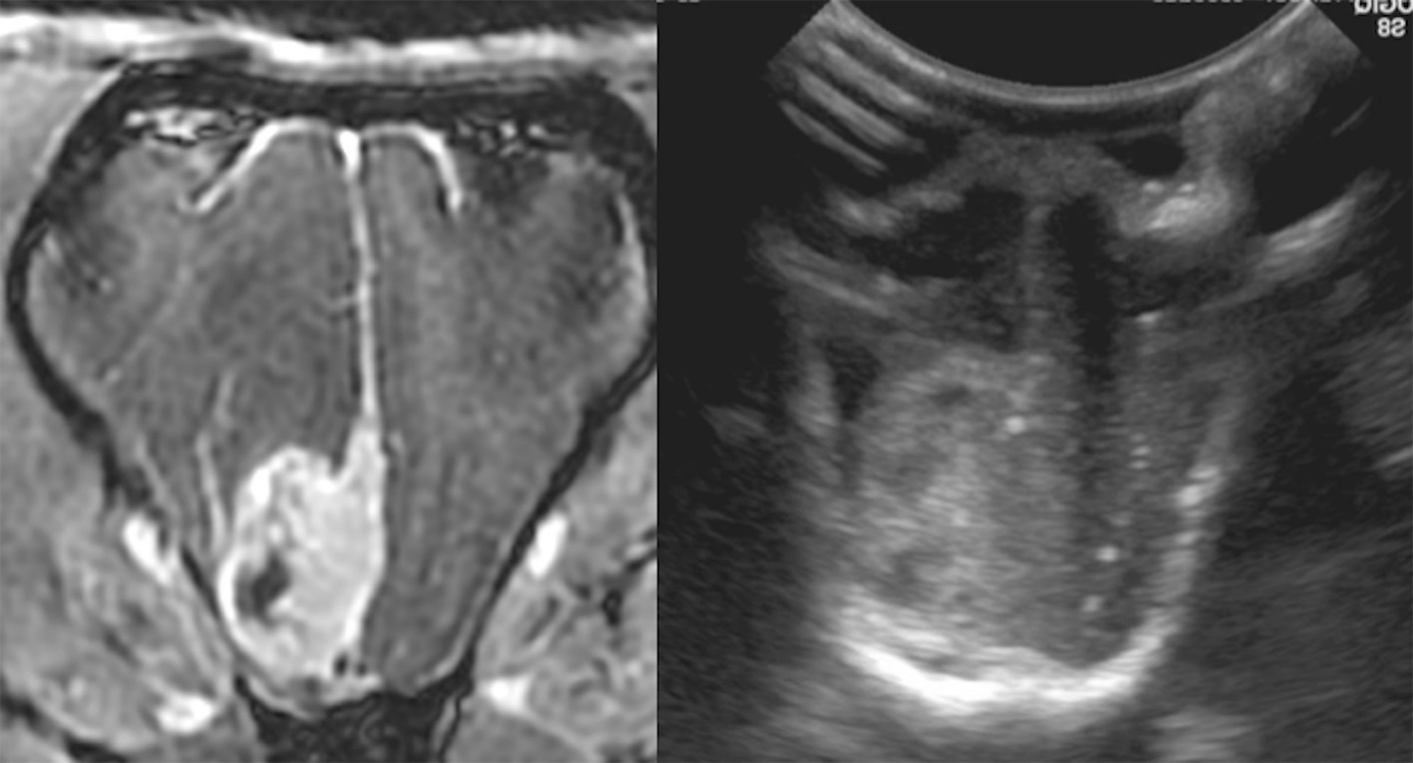
Case 29. There is an ovoid, sharply marginated, 1.46 × 0.81 cm (MRI)/1.49 × 0.85 cm (US), heterogeneous mass present to the right and adjacent to midline, which is largely hyperechoic to brain parenchyma, but also contains a few hypoechoic regions within. There is a mild leftward midline shift of the brain parenchyma as a result of this mass. Microscopic examination of this mass revealed a grade 1 transitional meningioma.

**Figure 5:**
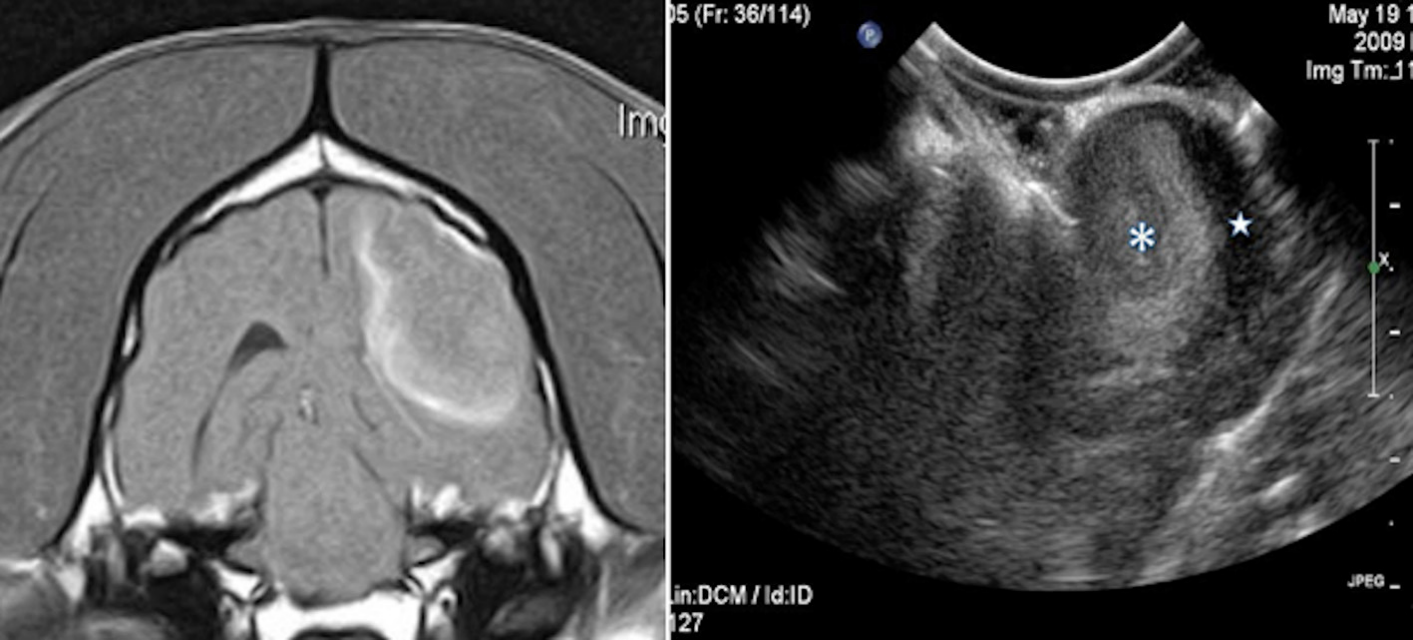
Case 1. Left, a transverse T1 + Contrast image of a meningioma. Right, a sagittal intraoperative ultrasound showing the mass () and adjacent lateral ventricle ().

All the tumors (29 canine and three feline patients) were very conspicuous, hyperechoic, and distinctly marginated during IOUS. All of the tumors correlated with preoperative imaging in location and estimated dimensions/volumes (Figures 2–5). In addition, data (Table 2) were available for the transverse plane for 20 of the canine tumors and statistical analysis revealed the mean difference between U/S and MRI height measurements was 0.027 cm with a standard deviation of 0.059 cm. This difference was not significant by a two-tailed paired *t*-test (*p* = 0.0559) with a power of 0.49 assuming an alpha level of 0.05. The sample size was sufficient to detect a difference of 0.05 cm with a power of 0.95 assuming the same standard deviation and alpha level. The mean difference between U/S and MRI width measurements was 0.013 cm with a standard deviation of 0.076 cm. This difference was not significant by a two-tailed paired *t*-test (*p* = 0.4345) with a power of 0.11 assuming an alpha level of 0.05 (7). The sample size was sufficient to detect a difference of 0.065 cm with a power of 0.95 assuming the same standard deviation and alpha level. The mean difference between U/S and MRI product measurements was 0.056 cm^2^ with a standard deviation of 0.128 cm^2^. This difference was not significant by a two-tailed paired *t*-test (*p* = 0.0647) with a power of 0.46 assuming an alpha level of 0.05. The sample size was sufficient to detect a difference of 0.11 cm^2^ with a power of 0.95 assuming the same standard deviation and alpha level.

**Figure 6:**
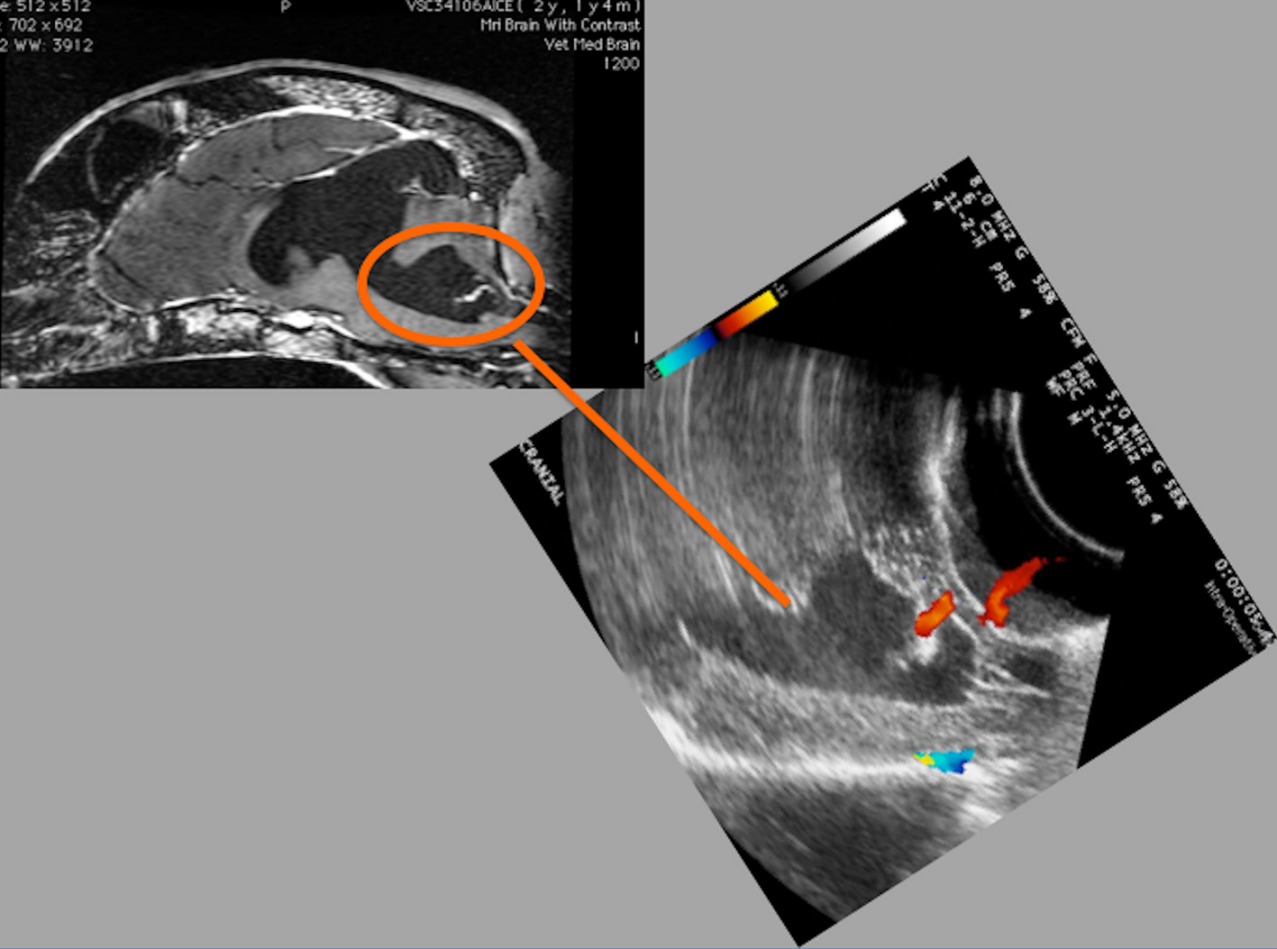
Case 21. Left, T1 sagittal MRI. Right, accompanying IOUS image. There is moderate fluid dilation of the fourth ventricle, measuring up to 1.8 cm in height (orange circle on MRI with line connecting to corresponding area on ultrasound image). There is additional fluid dilation present dorsal to the colliculi. Moderate blood flow is seen on color Doppler integration surrounding the dilated fourth ventricle. The surrounding brain parenchyma is mildly hyperechoic.

**Figure 7:**
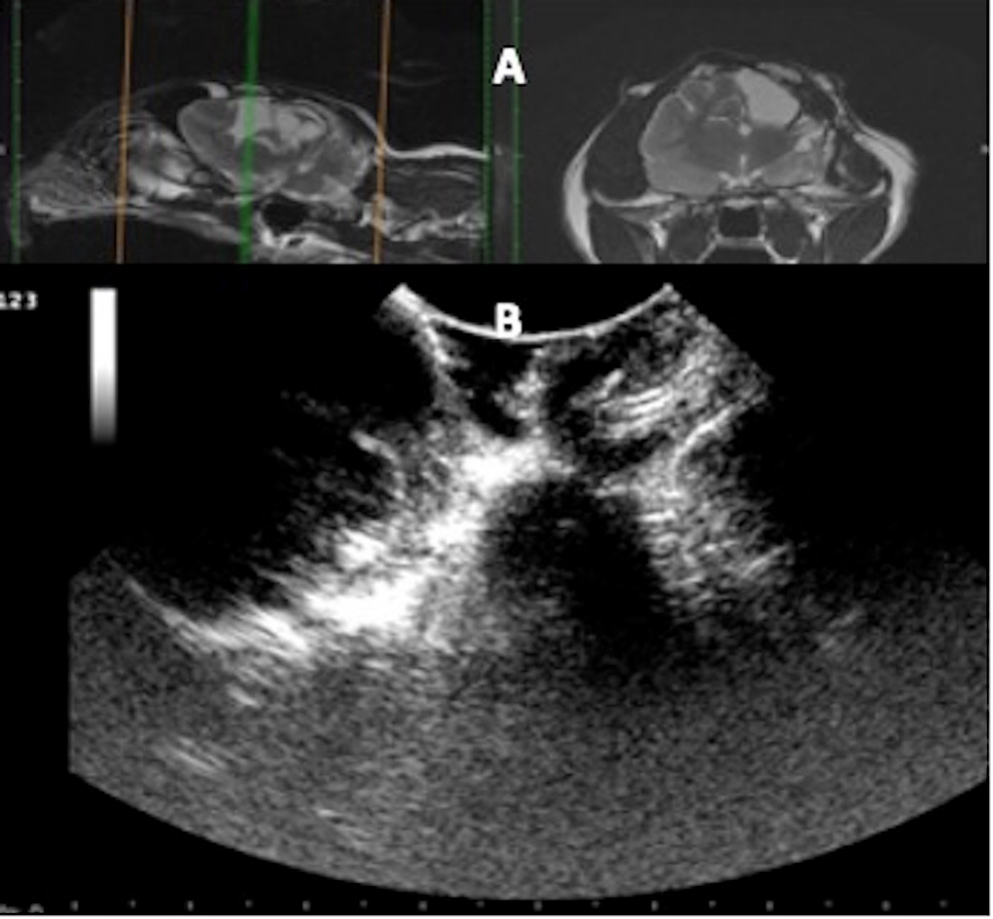
Case 36. **(A)** Sagittal and transverse magnetic resonance images of a cat that suffered a skull fracture 6 months before presentation and presented for a sudden onset of generalized seizures. The injury site consisted of fluid-filled areas and devitalized cortical tissue. **(B)** At surgery, the ultrasound identified the fluid-filled areas and adjacent unaffected cerebral tissue.

**Figure 8:**
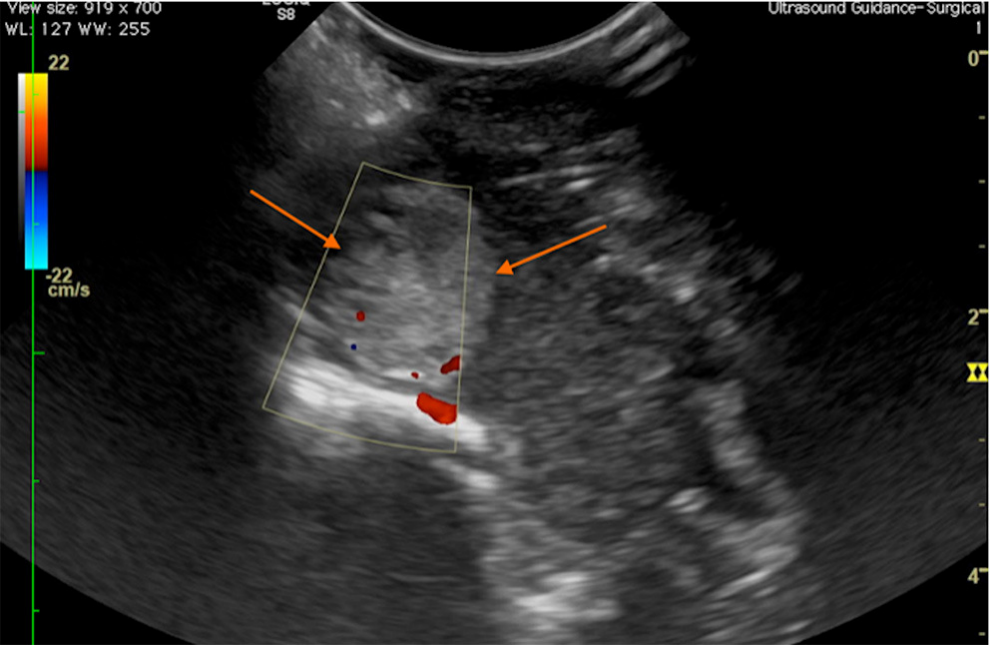
Case 28. Transverse intraoperative ultrasound view of a frontal meningioma (arrows) in a canine patient. The Doppler shows that the major arterial blood supply to the mass is at the ventral aspect, in the area of the floor of the calvarium.

**Figure 9:**
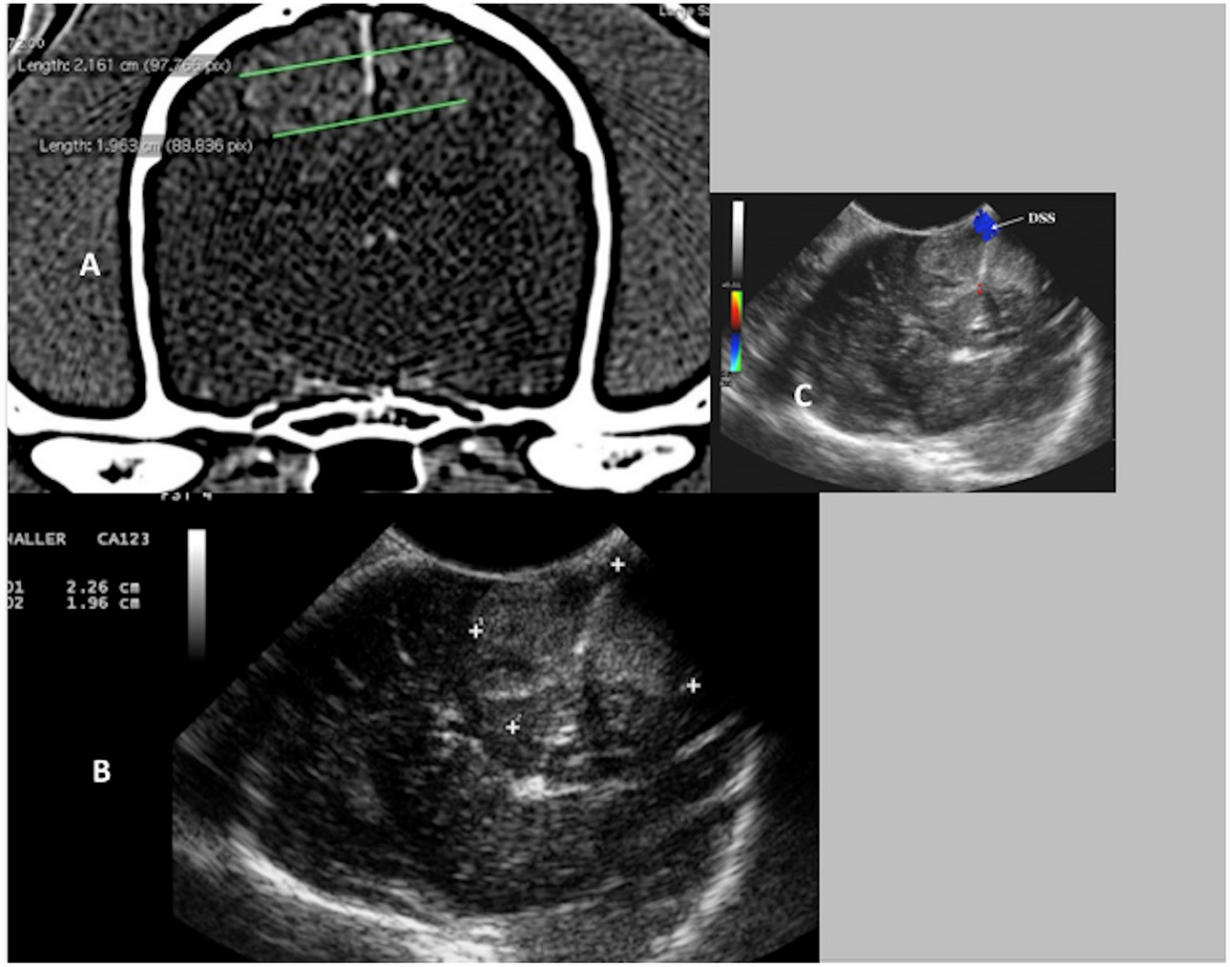
Case 17. **(A)** Transverse CT image of a canine brain showing bilateral falcine meningiomas. **(B)** Transverse intraoperative ultrasound image of the bilateral falcine masses. Note the very close similarities in measurements on the CT and the ultrasound images (CT 2.16 and 1.96 cm vs. US 2.26 and 1.96 cm). **(C)** Transverse intraoperative ultrasound with Doppler, demonstrating the position of the dorsal sagittal sinus (DSS).

**Figure 10:**
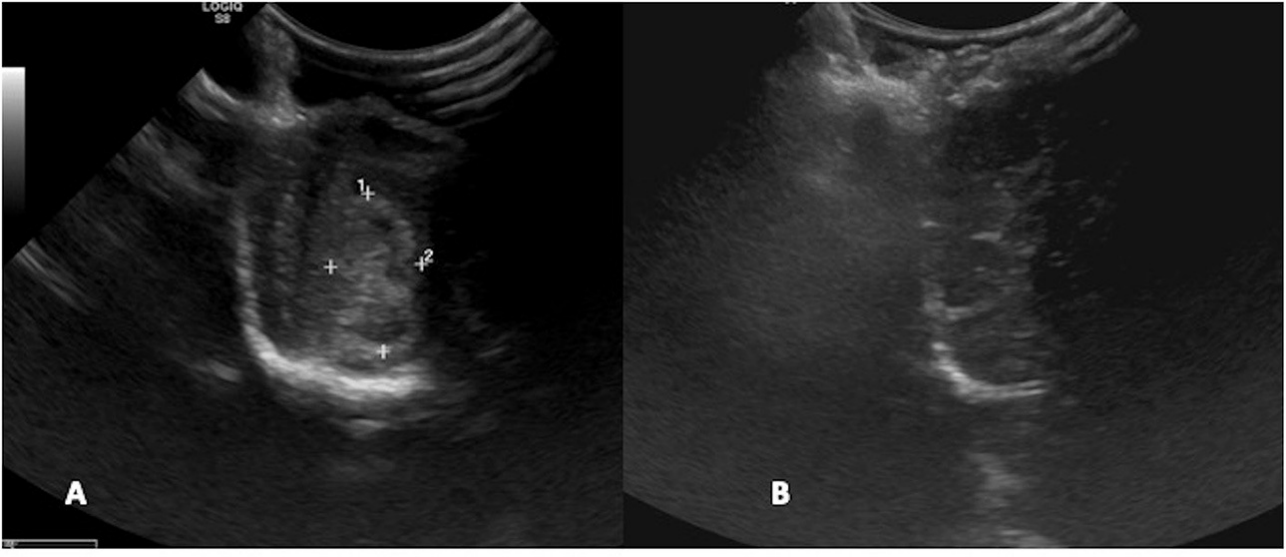
Case 29. **(A)** Intraoperative ultrasound appearance of frontal meningioma before removal and **(B)** following gross tumor resection.

**Figure 11:**
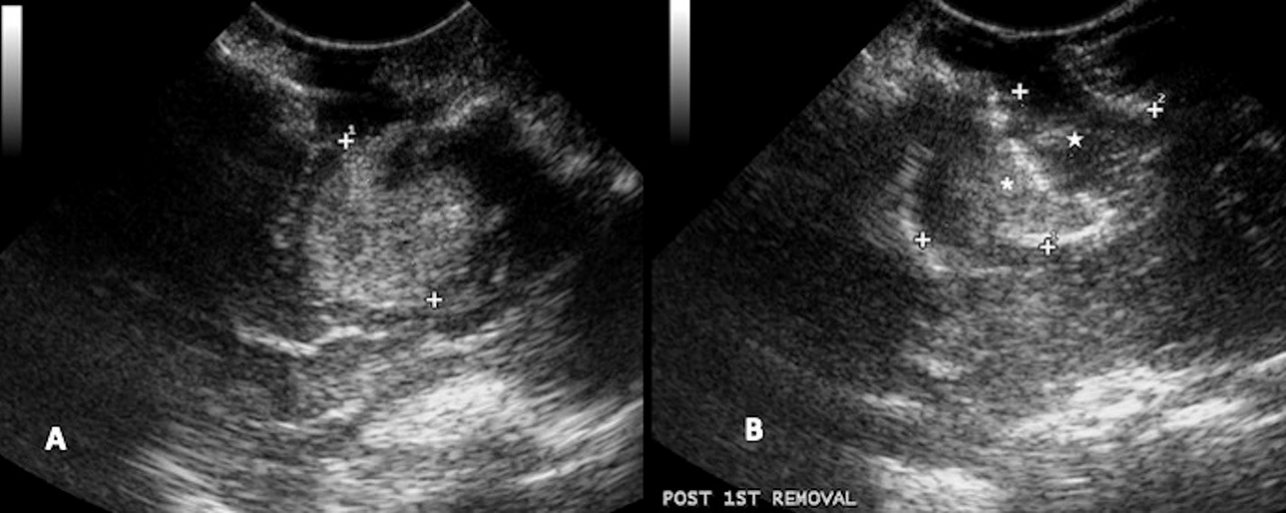
Case 24. **(A)** Intraoperative ultrasound appearance of high-grade glioma before removal and **(B)** following partial removal. In B, the intraoperative ultrasound clearly shows the portion of the tumor removed (star) and the remaining tumor (asterisk).

**Figure 12:**
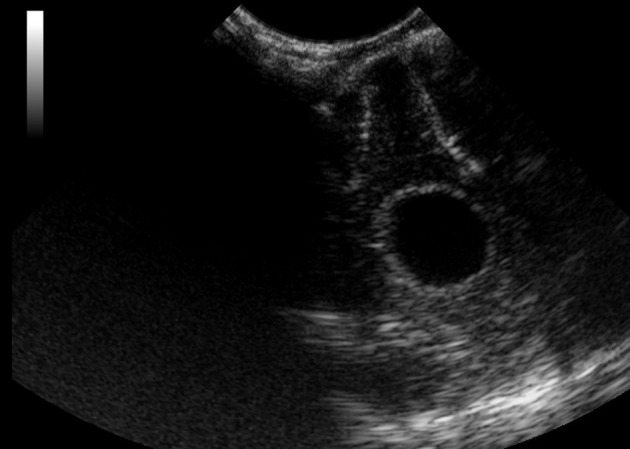
Case 6. Intraoperative ultrasound view of a suspected abscess that was later diagnosed as *isolated angiitis* by histopathology following a post-mortem examination. The mass was located in the frontal lobe and appeared hypoechoic on ultrasound imaging.

In non-tumor cases (four canine patients and two feline patients), the IOUS images gave an accurate depiction of the neuroanatomical structures demonstrated on preoperative imaging (Figure 6). In the feline patient with a skull fracture, the IOUS images showed a clear distinction between normal and damaged structures of the brain (Figure 7). When postoperative imaging was performed in tumor patients, the CT or MRI images and the IOUS images correlated with the changes seen after tumor removal as reported by the radiologist. Real-time assessment of progress in mass removal and the proximity of the larger vessels when utilizing the Doppler mode on the ultrasound unit were evident with IOUS (Figures 8, 9). In Figure 10, the initial IOUS is compared to the IOUS image following gross total resection of a meningioma. In Figure 11, the initial IOUS is compared to images acquired during the procedure in the removal of a high-grade glioma. Figure 12 demonstrates the hypoechoic IOUS appearance of a mass originally diagnosed as an abscess, but later described as isolated angiitis on post-mortem examination. The more hyperechoic borders of the mass are clearly seen in this image.

## Discussion and Conclusions

Neurosurgeons had no difficulty utilizing the IOUS probe. In all patients, a radiologist assisted in surgery by operating the ultrasound unit and interpreting the images in real time, allowing the surgeons to focus on the procedures and have real-time assessment of progress in mass removal. With the increased experience in using the IOUS in conjunction with the Cavitron Ultrasonic Surgical Aspirator (CUSA) device for mass removal, our surgical approaches are more easily designed with preoperative planning. The use of the IOUS aids in the design and conciseness of the surgical approach and often reduces the extent of the opening into the cranial vault. This perhaps reduces surgical time by real-time image guidance in resection of the mass. The visual differences in normal cerebral tissue and tumor, especially gliomas, are often subtle; however, our study demonstrates a clear distinction in echogenicity of the two tissues with IOUS. This is a strong advantage in maximizing tumor removal with minimal resection of normal surrounding cerebral tissue. In addition, real-time identification of the blood supply to the tumor, bleeding at the operative site, and bleeding as a result of the biopsy are very beneficial at the time of surgery.

Some other methods of intracranial surgery imaging include frame-based stereotactic biopsy (7), intraoperative MRI (8), intraoperative biological staining (9), and the intraoperative navigation systems (10). Each of these are unique with certain advantages and disadvantages. Of these others, only the intraoperative MRI affords the surgeon a real-time image without any concern about intraoperative brain shift. The frame-based stereotactic biopsy technique is only utilized for biopsy and not for gross tumor resection. The intraoperative staining can provide better visualization of the mass at the beginning of resection, but visualization may be skewed when hemorrhage around the tumor places stain in the surgical field over the surrounding tissues. Some intraoperative staining techniques require special and very expensive light filters in the operating room. For deeper tumors, as is commonly encountered with temporal lobe gliomas, direct visualization without ultrasound is difficult and the staining would be of limited assistance. The neuronavigation system used by some veterinary neurosurgeons (10) is very accurate based on the pre-biopsy MRI; however, this too is mainly used for biopsy and not gross total resection. For the purpose of gross total resection of intracranial masses, avoiding issues with brain shift, and visualization of surrounding vasculature, the authors have found IOUS to be advantageous in veterinary intracranial surgery.

## Data Availability Statement

The original contributions presented in the study are included in the article/supplementary material, further inquiries can be directed to the corresponding author.

## Ethics Statement

Ethical review and approval was not required for the animal study because these were clinical patients and only standard procedures/equipment were used and all had full client consent for the procedures performed. Written informed consent was obtained from the owners for the participation of their animals in this study.

## Author Contributions

AS: Is a faculty neurosurgeon, initiated the technique incorporating IOUS into the intracranial surgery protocol, performed a majority of the surgical procedures, collected the data, and drafted the manuscript. AML: The chief radiologist associated with the project and conducted much of the IOUS imaging and reporting of findings, plus assisted in drafting the manuscript. STK: A neurosurgery resident present and a participant in many of the surgeries. CT: Was a diagnostic imaging resident and performed several of the imaging procedures and selected several of the images shown in the figures. MAS: Was a diagnostic imaging resident and performed several of the imaging procedures. RWW: Performed and interpreted the statistical analysis. MBB: Is a faculty neurosurgeon and performed and assisted in several of the surgical procedures.

## Conflict of Interest

The authors declare that the research was conducted in the absence of any commercial or financial relationships that could be construed as a potential conflict of interest.

## Publisher's Note

All claims expressed in this article are solely those of the authors and do not necessarily represent those of their affiliated organizations, or those of the publisher, the editors and the reviewers. Any product that may be evaluated in this article, or claim that may be made by its manufacturer, is not guaranteed or endorsed by the publisher.

## Footnotes

^1^Signa Excite HDx 3T magnet, GE, Milwaukee, WI.

^2^LightSpeed 64-slice VCT, GE, Milwaukee, WI.

^3^BioSound Esaote MyLab50 ultrasound machine (Esaote North America, Indianapolis, IN) and microconvex probe with available frequencies ranging from 5 to 8 MHz.

^4^GE LOGIQ S8 ultrasound machine (General Electric, Boston, MA) with a C3-10-D Broad Spectrum microconvex transducer (6–10 MHz); 11L-D linear transducer (9–12 MHz).

^5^CIV-Flex Transducer Cover, Civco, Kalona, IA.

^6^Integra CUSA NXT, Soma Technology, Bloomfield, CT.

^7^SAS for Windows v9.4, SAS Institute, Inc., Cary, NC.
